# Effects of Titanium Dioxide Nanoparticles on the *Hprt* Gene Mutations in V79 Hamster Cells

**DOI:** 10.3390/nano10030465

**Published:** 2020-03-05

**Authors:** Alena Kazimirova, Naouale El Yamani, Laura Rubio, Alba García-Rodríguez, Magdalena Barancokova, Ricard Marcos, Maria Dusinska

**Affiliations:** 1 Department of Biology, Faculty of Medicine, Slovak Medical University, 833 03 Bratislava, Slovakia; magdalena.barancokova@szu.sk; 2 Health Effects Laboratory, Department of Environmental Chemistry, NILU-Norwegian Institute for Air Research, N-2027 Kjeller, Norway; ney@nilu.no; 3 Nanobiology Laboratory, Department of Natural and Exact Sciences, Pontificia Universidad Católica Madre y Maestra, PUCMM, 80677 Santiago de los Caballeros, Dominican Republic; l.rubio@ce.pucmm.edu.do; 4 Department of Genetics and Microbiology, Faculty of Biosciences, Universitat Autònoma de Barcelona, 08193 Cerdanyola del Vallès (Barcelona), Spain; albagr.garcia@gmail.com (A.G.-R.); ricard.marcos@uab.es (R.M.); 5 Consortium for Biomedical Research in Epidemiology and Public Health (CIBERESP), Carlos III Institute of Health, 28029 Madrid, Spain

**Keywords:** titanium dioxide nanoparticles, V79 cells, genotoxicity, *Hprt*

## Abstract

The genotoxicity of anatase/rutile TiO_2_ nanoparticles (TiO_2_ NPs, NM105 at 3, 15 and 75 µg/cm^2^) was assessed with the mammalian in-vitro Hypoxanthine guanine phosphoribosyl transferase (*Hprt*) gene mutation test in Chinese hamster lung (V79) fibroblasts after 24 h exposure. Two dispersion procedures giving different size distribution and dispersion stability were used to investigate whether the effects of TiO_2_ NPs depend on the state of agglomeration. TiO_2_ NPs were fully characterised in the previous European FP7 projects NanoTEST and NanoREG2. Uptake of TiO_2_ NPs was measured by transmission electron microscopy (TEM). TiO_2_ NPs were found in cytoplasmic vesicles, as well as close to the nucleus. The internalisation of TiO_2_ NPs did not depend on the state of agglomeration and dispersion used. The cytotoxicity of TiO_2_ NPs was measured by determining both the relative growth activity (RGA) and the plating efficiency (PE). There were no substantial effects of exposure time (24, 48 and 72 h), although a tendency to lower RGA at longer exposure was observed. No significant difference in PE values and no increases in the *Hprt* gene mutant frequency were found in exposed relative to unexposed cultures in spite of evidence of uptake of NPs by cells.

## 1. Introduction

Nano-sized or ultrafine titanium dioxide particles (TiO_2_ NPs) are among the most widely used nanomaterials. TiO_2_ is a poorly soluble particulate material with numerous applications such as food colorant or white pigment in the production of paints, paper, plastics, ink and welding rod-coating material. TiO_2_ NPs (<100 nm) are increasingly used in other industrial products, such as cosmetics, skin care products (in sunscreens, as an ultraviolet blocking agent), toothpaste, and pharmaceuticals [1,2,3,4]. It can even be used as a food additive, for example to whiten skimmed milk [5]. Therefore, potential widespread exposure may occur during manufacturing and use [6].

Whether TiO_2_NPs represent any hazard to humans is a question addressed by various regulatory agencies. Genotoxicity studies of TiO_2_NPs have been widely performed detecting different types of DNA damage such as strand breaks and various DNA lesions (using mostly the comet assay), gene mutations in bacteria and in mammalian cells, as well as chromosomal damage representing possible clastogenic or aneugenic effects. However, in-vivo and in-vitro studies have reported conflicting results; some indicate that TiO_2_ NPs are genotoxic [6,7,8,9], whereas others give negative results [8,10,11,12]. This inconsistency is related to the different particle types used, with different NP sizes and physico-chemical properties, NP dispersion and exposure conditions, as well as to the use of different cell culture media, cellular models, and test methods [13,14,15,16]. Most of genotoxic effects are seen in cells derived from the respiratory, and the circulatory systems. Where internal exposure of the lungs can occur, there is a possibility that TiO_2_ NP may exert genotoxic effects, most probably through secondary mechanisms (e.g. oxidative stress); however, direct interaction with the genetic material cannot be excluded. Overall, the studies indicating that TiO_2_ NPs are genotoxic outweigh the studies that state otherwise. According to that, TiO_2_ NPs can be treated as potentially hazardous compounds [5] consistent with the fact that TiO_2_ itself is classified as a class 2B carcinogen [17].

The mammalian gene mutation tests belong to the set of assays recommended by the regulatory bodies and, in nanomaterial genotoxicity testing, they are preferred since the Ames test is not suitable due to the size of bacteria (comparable with NPs themselves) and the fact that the bacterial wall limits significantly the uptake of NPs [14,18]. The most commonly used target genes for measuring the induction of mutations in mammalian cells are the thymidine kinase (*Tk*) and the hypoxanthine guanine phosphoribosyl transpherase (*Hprt*) genes. Specifically, the *Hprt* mutation assay has already been successfully applied to the evaluation of different nanomaterials [6,18,19,20,21]. 

With regard to studies using TiO_2_ NPs to induce *Hprt* mutants, previous studies have already been reported, where positive effects were observed in the WIL2-NS human B-cell lymphoblastoid cell line (24 h exposure of 130 µg/mL UF-TiO_2_) [6], and in V79-4 hamster cells (2 h of short-term treatment of 20 and 100 µg/mL anatase TiO_2_ NPs [7]. Interestingly, negative results were obtained with anatase TiO_2_ NPs (10–40 µg/mL) in a long-term (60 days) exposure experiment. In that case, Chinese hamster ovary cells (CHO-K1) cells appear to adapt to chronic exposure to TiO_2_ NPs and detoxify the excess of reactive oxygen species (ROS), possibly through an up-regulation of super oxide dismutase (SOD), in addition to reducing particle uptake [10]. 

In this context, the aim of our work is to investigate whether TiO_2_ NPs induce mutagenic effect in the *Hprt* gene, and whether this effect depends on the dispersion procedure used, e.g. on different states of agglomeration.

## 2. Material and Methods

### 2.1. Cells

V79-4 adherent hamster cells isolated from the lung of a normal Chinese hamster (male), were purchased from European Collection of Authenticated Cell Cultures (ECACC, catalogue number 86041102). Cells were cultured in Dulbecco’s minimal essential medium (DMEM) D6046 (Sigma, Steinheim, Germany) supplemented with 10% (v/v) heat-inactivated fetal bovine serum (FBS, Gibco, Grand Island, NY, USA) and 1% (v/v) penicillin-streptomycin (Gibco) at 37 °C in a 5% CO_2_ humidified atmosphere. Cells were thawed and sub-cultured 2–4 times before use in the experiments, at an initial density of 2 × 10^5^ cells/mL in vented T-75 cm^2^ flasks. Cultures were maintained with density not exceeding 1 × 10^6^ cells/mL at the time of passage. Cells were seeded 24 h to reach 50–70% confluence before exposure to test substance. Trypan blue assay was used to check cell viability after trypsinization of cells. 

### 2.2. Nanoparticle Characterization, Dispersion and Cell Exposure

TiO_2_ NP (NM-105), an anatase/rutile nanopowder of nominal size 21 nm (15–60 nm), was received from the EU Joint Research Centre (Ispra, Italy). It was manufactured by Evonik (Essen, Germany), and marketed as Aeroxide TiO_2_ P-25. TiO_2_ NPs were fully characterised in previous EU projects [13,22], and results are summarised in Table 1.

For the treatment of cells we used TiO_2_ NP dispersed by two different procedures, either with or without serum in stock solution. This can permit investigations on how the state of aggregation/agglomeration and stability of the dispersion could influence TiO_2_ NP cytotoxicity and genotoxicity. 

#### Dispersion Procedure DP1

Stock suspensions of TiO_2_ NPs at 5 mg/mL were freshly prepared for each experiment, using the dispersion procedure DP1 developed as part of the FP7 project NanoTEST. For 1 mL of stock suspension, 5 mg of TiO_2_ NPs mixed with 1 mL of 10% fetal bovine serum (FBS, Gibco) in PBS (phosphate buffered saline) in a glass tube was sonicated using an ultrasonic probe sonicator (Labsonic, Sartorius, Gottingen Germany) at 100 W for 15 min on ice/water. This suspension was added to cell culture medium. Serial dilutions were made in cell culture medium to obtain the full range of NP suspensions, from 3 to 75 µg/cm^2^, which were then immediately added to cells.

#### Dispersion Procedure DP2

In DP2, 20 mg of TiO_2_ NPs was suspended in 10 mL of culture medium with 15 mM HEPES buffer and without FBS (the procedure developed at University Paris Diderot France [13]). The suspension was sonicated using the ultrasonic probe sonicator at 60 W for 3 min on ice/water, vortexed for 10 s, and within 2 min of sonication—aliquoted and stored at −20 °C for further use. TiO_2_ NP suspension aliquots were thawed just before use, vortexed for 10 s, sonicated at 60 W for 1 min on ice/water and added to cell culture medium. Serial dilutions were made in cell culture medium to obtain the full range of TiO_2_ NP suspensions from 3 to 75 µg/cm^2^, which were then immediately added to cells.

### 2.3. Extrinsic Properties of TiO_2_ NPs

Particle size, size distribution, state of agglomeration and stability of TiO_2_ NPs, both in stock solution as well as in culture medium, were characterized by Nanoparticle Tracking Analysis (NTA) using NanoSight NS 500 (NanoSight Limited, Netherhampton, Salisbury, UK). Table 2 shows size, agglomeration state and stability in culture medium measured by Dynamic Light Scattering DLS [22].

### 2.4. Cellular Uptake

Cellular uptake of TiO_2_ NPs was measured by transmission electron microscopy (TEM). V79-4 cells were grown on 6-well plates at a density of 1.75 × 10^5^ cells/well. Cells were exposed to TiO_2_ NPs dispersed according to DP1 and DP2 (3, 10, 30 μg/cm^2^) for 24 h. At the end of the exposure time, cells were centrifuged, fixed in 2.5% (v/v) glutaraldehyde (EM grade, Merck, Darmstadt, Germany) and 2% (w/v) paraformaldehyde (EMS, Hatfield, PA, USA) in 0.1 M cacodylate buffer at pH 7.4 (PB, Sigma-Aldrich, Steinheim, Germany), and processed following conventional procedures, as previously described [23]. Samples were first post-fixed with osmium tetroxide, dehydrated in acetone, later embedded in Epon, and finally polymerized at 60 °C, and cut with an ultramicrotome Leica EM UC6 using a diamond knife and mounted on copper grids. Before image acquisition, sections were stained using uranyl acetate and Reynolds lead-citrate solutions. All images were examined using a JEOL 1400 (JEOL LTC, Tokyo, Japan) TEM at 120 kV equipped with a CCD GATAN ES1000W Erlangshen camera.

### 2.5. Relative Growth Activity (RGA)

RGA measurements are based on cell proliferation activity of the cells during a period of treatment or after treatment with the tested compound. Cells were seeded at concentration 1 × 10^5^ cells per well on 12-well plates in 2 mL culture medium and were kept for 24 h under standard conditions at 37 °C in a 5% CO_2_ humidified atmosphere. Cells were then exposed to different concentrations (ranging from 0.12 to 75 μg/cm^2^) of TiO_2_ NPs lasting for 24, 48, and 72 h. Untreated cells, just with cell culture medium, were used as a negative control and hydrogen peroxide (H_2_O_2_, 100 µM, 5 min in PBS) was used as a positive control. Just after exposure, medium was removed from the culture and cells were washed with PBS, trypsinized, and re-suspended in 1 mL of medium. Finally, 10 μL of the final cell suspension was mixed with 10 μL of 0.4% trypan blue (Life Technologies, OR, USA) to determine the percentage of viable cells (unstained) and stained cells with damaged membranes. This determination was carried out using a Countess™ Automated Cell Counter (Invitrogen). RGA was calculated as already published [24].

### 2.6. Plating Efficiency (PE)

To determine the potential cytotoxic effects of the treatment, after 24 h exposure of V79-4 cells to TiO_2_ NPs, they were washed, trypsinized and counted, as described above. After that, 50 cells per well were inoculated in 6-well plates (for each concentration tested one plate was used) and incubated at 37 °C for 7 days. Untreated cells, just with cell culture medium, were used as a negative control. Finally, cells were stained by using 1% methylene blue (Sigma) and the number of resulting colonies was counted manually. PE values were calculated according to the formula:

$$PE\;\left(\%\right)=\frac{number\;of\;colonies\;in\;exposed\;cultures}{number\;of\;colonies\;in\;unexposed\;cultures}\times 100\%$$

### 2.7. Hprt Mammalian Gene Mutation Assay

The mammalian in vitro *Hprt* gene mutation test was performed according to the OECD Guidelines for the Testing of Chemicals 476 [25]. V79-4 cells were cultured in 100 mm diameter Petri dishes; 1 × 10^6^ cells were inoculated per dish in 10 mL medium in duplicate for each concentration, and incubated at 37 °C. On the following day, the cells were exposed to TiO_2_ NPs for 24 h, at concentrations from 3 to 75 μg/cm^2^.

Untreated cells cultured in medium for 24 h were used as negative control and cells treated for 3h with 0.1 mM methyl methanesulfonate (MMS; Sigma), served as the positive control.

After exposure, the medium was removed, and cells were washed, trypsinized and re-suspended in 2 mL of medium. They were then seeded in 100 mm diameter Petri dishes at 3 × 10^5^ cells/dish, 3 dishes per concentration. Cells were grown for 8 days, during which they were subcultured three times; duplicate samples were taken at 6 and 8 days after treatment for analysis of mutant frequencies. To detect mutants, cells were inoculated in 100 mm diameter Petri dishes at 2 × 10^5^ cells/dish, 5 dishes per sample giving a total of 10^6^ cells per sample and grown in medium containing 6-thioguanine (Sigma) at 5 μg/mL for 10 days to form colonies. 6-Thioguanine is an analogue of guanine, toxic to cells with functioning *Hprt* gene, and so only *Hprt^−^* cells survive. Mutant colonies were counted manually after staining with 1% methylene blue; only colonies with at least 50 cells were counted.

For each of the two harvests (6 and 8 day duplicate samples), the frequency of surviving cells was assessed using the PE assay, as described above. Treated and untreated cells were seeded in 6-well plates at 50 cells per well, 1 plate per concentration, and incubated for 7 days at 37 °C to form colonies. Cell viability was calculated for each mutant harvest on the basis of the number of colonies as a percentage of the number of inoculated cells. The mutant frequency was determined as previously described [24].

### 2.8. Statistical Analysis

One way analysis of variance ANOVA test was used, followed by Dunnett´s multiple comparison test for the post hoc analysis. Prism 7.0 (GraphPad Software, San Diego, CA, USA) and Microsoft Excel 2013 were used for statistics and mathematical analysis. Differences with *P* < 0.05 were considered statistically significant. 

## 3. Results

### 3.1. TiO_2_ Characterization, Extrinsic Properties

**Figure 1:**
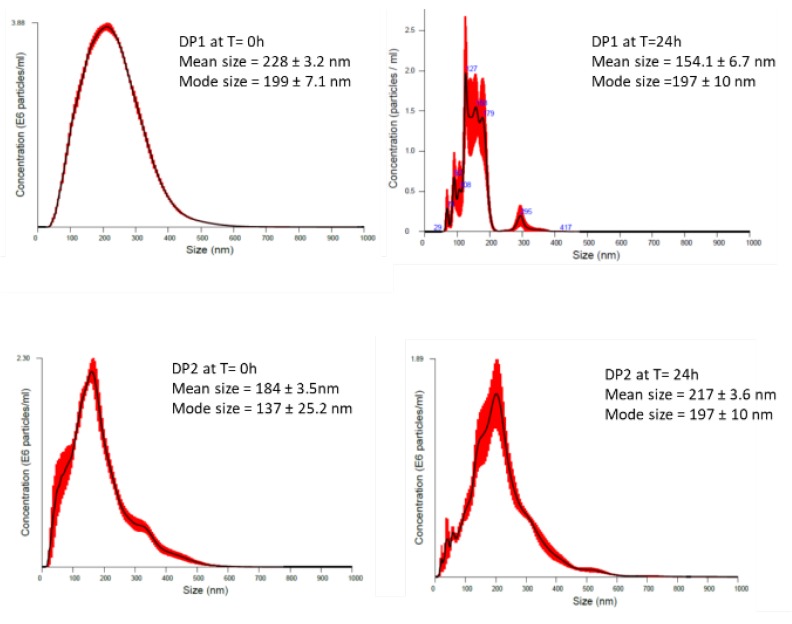
Particle size distribution obtained by (NTA) of TiO_2_ NPs using the two proposed dispersion procedures (DP1 and DP2) in culture medium at 0 and 24 h. The black line is the mean distribution and the red filling represent standard errors between captured videos.

As NPs change their properties depending on the surrounding environment, we also measured extrinsic properties of TiO_2_ NPs. We also aimed to identify whether size and stability of dispersion can influence the potential effect. The size distribution, state of agglomeration and stability of the tested TiO_2_ NPs were analysed in culture medium before the treatment and immediately after the treatment (times 0 and 24 h) by using NTA measured by NanoSight NS 500. The average size of the TiO_2_ NPs in DMEM at time 0, was 228 ± 3.2 nm, and 184 ± 3.5 nm, for DP1 and DP2, respectively. After 24 h, the mean size of TiO_2_ NPs prepared by DP1 was 154.1 ± 6.7, while TiO_2_ NPs prepared by DP2 had an average size of 217 ± 3.6 showing relatively stable dispersion for both DPs, as showed in Figure 1. After 24 h, the TiO_2_ NPs dispersion DP2 was similar to time 0 h, but when we compare the concentration of particles per mL between time 0 and 24 h, a decrease in the concentration was observed. Extrinsic characteristics of size, size distribution, and the level of agglomeration/ aggregation of NPs in dispersions measured by DLS are described in Table 2.

### 3.2. Uptake of TiO_2_ NPs Measured by the TEM

**Figure 2:**
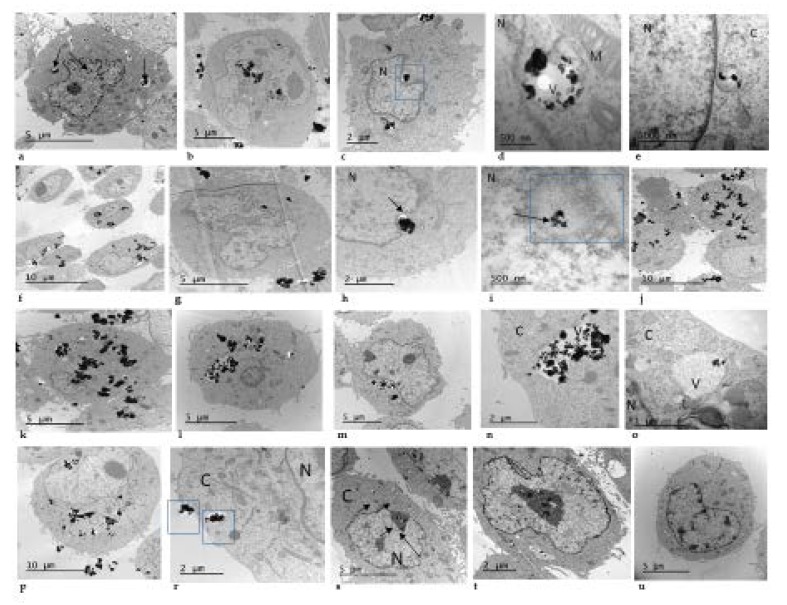
Representative transmission electron microscopy (TEM) figures of titanium dioxide TiO_2_ NM105 uptake by Chinese hamster lung fibroblast (V79-4) cells exposed to 3, 10 and 30 μg/cm^2^ of TiO_2_ NPs dispersed according to dispersion procedure 1 (DP1) and dispersion procedure 2 (DP2). DP1 3 μg/cm^2^ (**a**–**e**), DP1 10 μg/cm^2^ (**f**–**i**), DP1 30 μg/cm^2^ (**j**,**k**), DP2 3 μg/cm^2^ (**l**–**o**), DP2 10 μg/cm^2^ (**p**–**r**), DP2 30 μg/cm^2^ (**s**,**t**), Negative control untreated V79-4 cells (**u**). N = nucleus; C = cytoplasm; V = vesicle, M = mitochondrion.

The potential cell uptake of TiO_2_ NPs was investigated in V79-4 cells after exposures to 3, 10 and 30 µg/cm^2^ of the TiO_2_ NPs prepared using both dispersion procedures. Figure 2 shows that after 24 h of TiO_2_ NPs exposure they were taken up mostly as agglomerates and these were found in cytoplasm and vesicles. Agglomerates of TiO_2_ NPs were also detected in contact with the cell nucleus even when low concentrations of TiO_2_ NPs were used. It seems that the uptake of TiO_2_ NPs did not depend on the used dispersion since there was no difference in uptake of TiO_2_ NPs, whichever dispersion procedure was used.

### 3.3. Cytotoxic Effect of TiO_2_ NPs on V79-4 Cells

**Figure 3:**
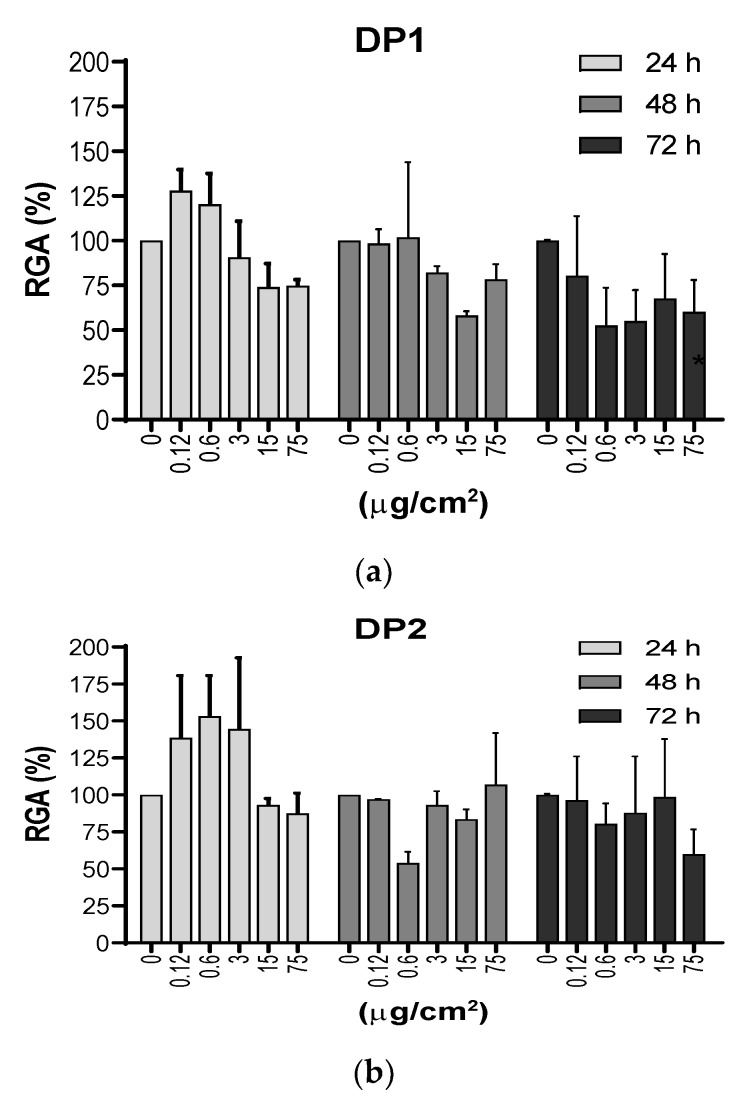
(**a**) and (**b**). Cytotoxic effects measured as the relative growth activity (RGA %) on V79-4 cells exposed to TiO_2_ NPs prepared using the two dispersion procedures (DP1 and DP2). Cells were treated with 5 concentrations (μg/cm^2^) of TiO_2_ NPs for 24, 48 and 72 h, and the cell numbers were counted at each time point immediately, following trypan blue staining. There were no statistical significances between exposed and unexposed cultures. Cytotoxicity of MMS was not been observed (RGA = 70%). Data are expressed as the means ± SEM of two parallel experiments, according to the used procedures.

An important endpoint for measuring the effect of NPs on cells is cytotoxicity. In our study the cytotoxicity of TiO_2_ NPs in V79-4 cells was measured by determining both the RGA and the PE values. RGA measures cytotoxicity in population of cells, while the PE gives information on individual cell toxicity. RGA values were determined as the ratio between the number of living cells, after exposures lasting for 24, 48 and 72 h, versus the number of living cells in the unexposed cultures. Figure 3 shows that, in general, TiO_2_ NPs exposures were not excessively toxic with rather more marked effects when DP1 was used. In addition, no significant effects of exposure time were seen, although there was a tendency to observe higher effects at longer exposure times.

**Figure 4:**
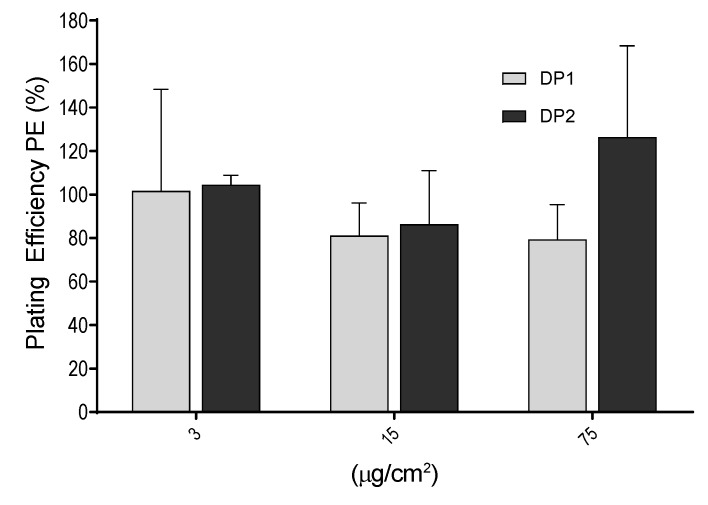
Cytotoxic effects of TiO_2_ NPs measured by the plating efficiency (PE %) in V79-4 cells. Bars represent cytotoxicity relative to 100% of untreated cells Data are expressed as the means ± SEM of two parallel seedings for plating efficiency, according to the used procedure. No statistical significances between exposed and unexposed cultures were observed.

The PE values were determined as the ratio of the number of colonies observed in the exposed cultures versus those observed in the unexposed cultures. Exposure lasted for 24 h and colonies were counted after 7 days of growth. As shown in Figure 4, no significant differences were observed between the negative control and each of the concentrations used (3, 15 and 75 µg/cm^2^). In addition, no differences in PE values were observed between the two dispersion procedures.

### 3.4. Mammalian Hprt Gene Mutation Assay, the Effect After TiO_2_ NPs Exposure

**Figure 5:**
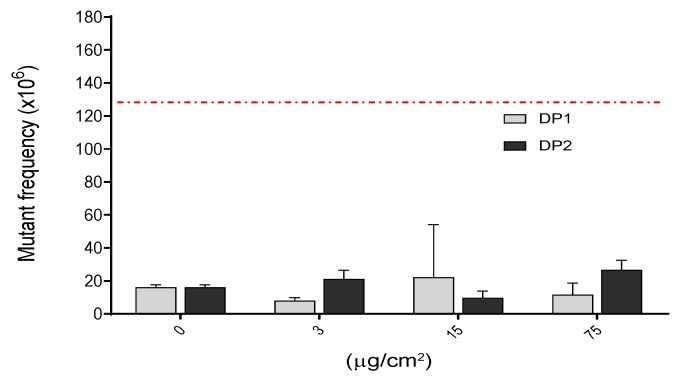
Induction of *Hprt* gene mutants after the exposure of V79-4 cells to different concentrations of TiO_2_ NPs for 24 h. There were no statistical significances between exposed and unexposed cultures. *Hprt* gene mutant frequency in treated cells with positive control MMS (0.1 mM, 3 h), which gave 131.6 ± 2.30 *Hprt* gene mutants. This value is indicated as a red-dashed line. Data are expressed as the means ± SEM of two parallel seedings for mutation frequency MF1 and MF2, according to the used procedure.

Genotoxicity is one of the most crucial effects that should be investigated in assessing safety of chemicals including NPs and it covers several genotoxicity endpoints, namely gene mutations, and structural and numerical chromosome aberrations. In our study we assessed the mutation potential of TiO_2_ NPs in V79-4 cells in two different experiments for each harvest point. As observed in Figure 5, there were no significant differences between the negative control and any of the three (3, 15 and 75 µg/cm^2^) concentrations used. This observed lack of mutagenic effects was independent of the dispersion used. In contrast, the positive control (MMS, 0.1 mM, 3 h) showed a clear induction of *Hprt* mutants, supporting the validity of the assay, and confirming the lack of mutagenic potential of the TiO_2_ NPs, at least under our experimental conditions.

## 4. Discussion

In-vitro toxicology data, based on well-designed experiments are required for risk assessment strategies designed for the testing of engineered nanomaterials. Until now, while in-vitro tests have been successfully applied in nanotoxicology studies, reference and quality standards are not always included: determination of physico-chemical properties, a range of appropriate controls (including stabilizer controls) and representative cell models, among other aspects, are crucially important.

Furthermore, physical processes in the preparation of the nanomaterials to be tested, such as dissolution, aggregation and sedimentation must be taken into consideration to better understand the mechanism of ENM toxicity [26]. In our study, the effects of TiO_2_ NPs were compared using two different DPs: one with serum in the stock solution and one without, in order to investigate whether the dispersion procedure and dispersion components could influence NP cytotoxicity and genotoxicity. In addition, we have evaluated the genotoxic potency by detecting their ability to induce gene mutations at the *Hprt* locus. It should be noted that the evaluation of the genotoxic potential of TiO_2_ NPs has been the subject of different reviews [16,27,28,29,30].

The *Hprt* gene mutation assay has been widely used in human biomonitoring and this target seems to be a valuable biomarker to determine the genotoxic/carcinogenic risk of exposures [31]. Accordingly, the mammalian gene mutation test is considered a surrogate in vitro marker for use in cancer risk assessment, together with the micronucleus assay. The use of the *Hprt* forward gene mutation assay allows the quantification of a wide set of genetic changes such as base substitutions, amplifications, or small deletions. This assay has already been used to determine the mutagenicity of TiO_2_ NPs in different types of cells [6,7,10,32]. Thus, in cultured WIL2-NS cells, a human B-cell lymphoblastoid cell line, ultrafine TiO_2_ particles (<100 nm in diameter) induced approximately 2.5-fold increases in the mutation frequency, in addition to significant toxicity [6]. However, negative results were obtained when TiO_2_ NPs were evaluated in Chinese hamster ovary (CHO-K1) cells subject to chronic exposures of up to 60 days [10]. In such cells no cytotoxic effects were apparent using the XTT (2,3-bis(2-methoxy-4-nitro-5-sulfophenyl)22H-tetrazolium-5-caboxyanilide), trypan-blue exclusion, and colony-forming assays for viability and, in addition, no variations in the frequency of *Hprt* mutations were reported. Finally, the *Hprt* assay has also been used in V79 cells to determine the mutagenic potential of TiO_2_ NPs, showing a clear dose-dependent effect [7,32]. This disparity in the obtained results would support the view that there are many factors affecting the outcome when the genotoxicity of NPs in general, and TiO_2_ NPs in particular, is evaluated. It is important to point out that, in spite of the reported contradictory data, TiO_2_ NPs are well taken up by mammalian cells, including V79 cells. In this case, our positive uptake findings have been confirmed by a recent study using flow cytometric analysis and TEM in the same cell line [32]. In this study TEM micrographs showed the internalization (confirmed by SEM/EDX analysis) of TiO_2_ NPs in the cytoplasm inducing ultra-structural changes such as swollen mitochondria and nuclear membrane disruption.

The ability of TiO_2_ NPs to produce gene mutants has also been tested using gene targets other than the *Hprt* gene. Thus, the mouse lymphoma assay targeting the *Tk* gene was used to determine the mutagenicity of TiO_2_ NPs, with negative results [33]. These negative results were obtained independently of the presence/absence of the microsomal S9 fraction in the culture medium.

As previously indicated, the *Hprt* assay has also been used to evaluate the genotoxic potential of other nanomaterials. Thus, by using silver nanomaterials it was shown that such NPs were mutagenic in V79-4 cells and, interestingly, this effect depended on their size [26]. On the other hand, amorphous silica NPs were evaluated for the detection of both *Hprt* mutants and ROS production in V79 cells, with negative results [34]. Furthermore, when multi wall carbon nanotubes were tested in the dose-range of 0.12 to 12 µg/cm^2^, significant cellular uptake was observed by using transmission electron microscopy. In addition, a clear concentration-dependent increase in the induction of *Hprt* mutants was seen together with a significant increase in the levels of intracellular reactive oxygen species [20]. Finally, this gene mutation assay has also been used to detect the mutagenic potential of nickel oxide NPs. In that case, a small but statistically significant increase in the frequency of *Hprt* mutations was observed for NiO NPs but only at one of the different tested doses [21].

From our results it appears that the dispersion procedure is not a factor modulating the genotoxicity of TiO_2_ NPs. Thus, there were no significant increases in the *Hprt* gene mutation frequency when the two different methods in our study were applied. These results would agree with those reported using the same TiO_2_ NPs, where no increases in the frequency of micronuclei in TK6 cells, rat bone marrow erythrocytes, or human lymphocytes were observed following three different dispersion procedures [35]. In the same study, using the comet assay, TiO_2_ NPs dispersed in a stable, non-agglomerated state were able to induce DNA strand breaks in human white blood cells, although no increases in levels of DNA oxidation were seen. The overall conclusion of that study is that NPs in an agglomerated state were unable to cause DNA damage. The observed differences in the results obtained with the different assays can be consequences of the differences in the mechanisms underlying the genotoxic effects detected by the different assays [35]. The dispersion procedure not only can affect genotoxicity but also toxicity. The levels of agglomeration/aggregation of NPs and their size distribution depends on the dispersion procedure, and on the use of serum in stock solution. In our case, DP1 using FBS gave a relatively stable dispersion of TiO_2_ NPs, while with the second procedure DP2, rapid formation of TiO_2_ NP agglomerates occurred in the testing medium as measured by DLS, as well as by TEM [13]. Our results show also a discrepancy in measurement of size distribution and stability of dispersion in exposure medium between NTA and DLS measurements, implying that DLS gives a more realistic measure of extrinsic properties of NPs, compared with NTA. 

Independently of the results obtained in this study, a procedure giving more stable dispersion should be preferred, so as to avoid false negative data that may be caused by the uptake difficulties associated with big agglomerations/aggregations.

## Figures and Tables

**Table 1:** Summary of primary physical and chemical properties of the used TiO_2_ NPs NM-105 [13].

Type of Characteristics	Properties of NM-105
Phase Shape of particles	White ultra-fine powder Irregular/ellipsoidal
Particle size (nm)	15–60
Crystal structure	Anatase/Rutile in ratio of 70:30 or 80:20
Surface area (m^2^/g)	61
Pore volume (mL/g)	0.13
Zeta-potential at pH 7 (mV)	−30.2
Chemical composition of particles	Ti, O
Ti purity of particles	>99%
Surface chemistry	Uncoated
Impurities of concern	Co (920 ppm), Fe (16 ppm)

**Table 2:** Average hydrodynamic diameters determined, by Dynamic Light Scattering (DLS), of the obtained TiO_2_ NPs stock dispersions [22].

Medium	TiO_2_ Stock Dispersion DP1	TiO_2_ Stock Dispersion DP2
DMEM +10% FBS	Bimodal distribution, 112 (± 20) nm and 296 (± 55) nm	752 (± 397) nm
Size stability after 48 h	Stable ~2 days 125 (± 27) nm and 366 (± 65) nm	Large Agglomerates

DMEM—Dulbecco’s minimal essential medium; FBS—fetal bovine serum.
